# Supplementary material for: PHOTOPERIOD 1 enhances stress resistance and energy metabolism to promote spike fertility in barley under high ambient temperatures
Source: Plant Physiol. 2025 Mar 26;197(4):kiaf118. doi: 10.1093/plphys/kiaf118 (PMC12002028; doi:10.1093/plphys/kiaf118)
Supplement: kiaf118_Supplementary_Data [file kiaf118_supplementary_data.zip › all_supplementary_materials.pdf]

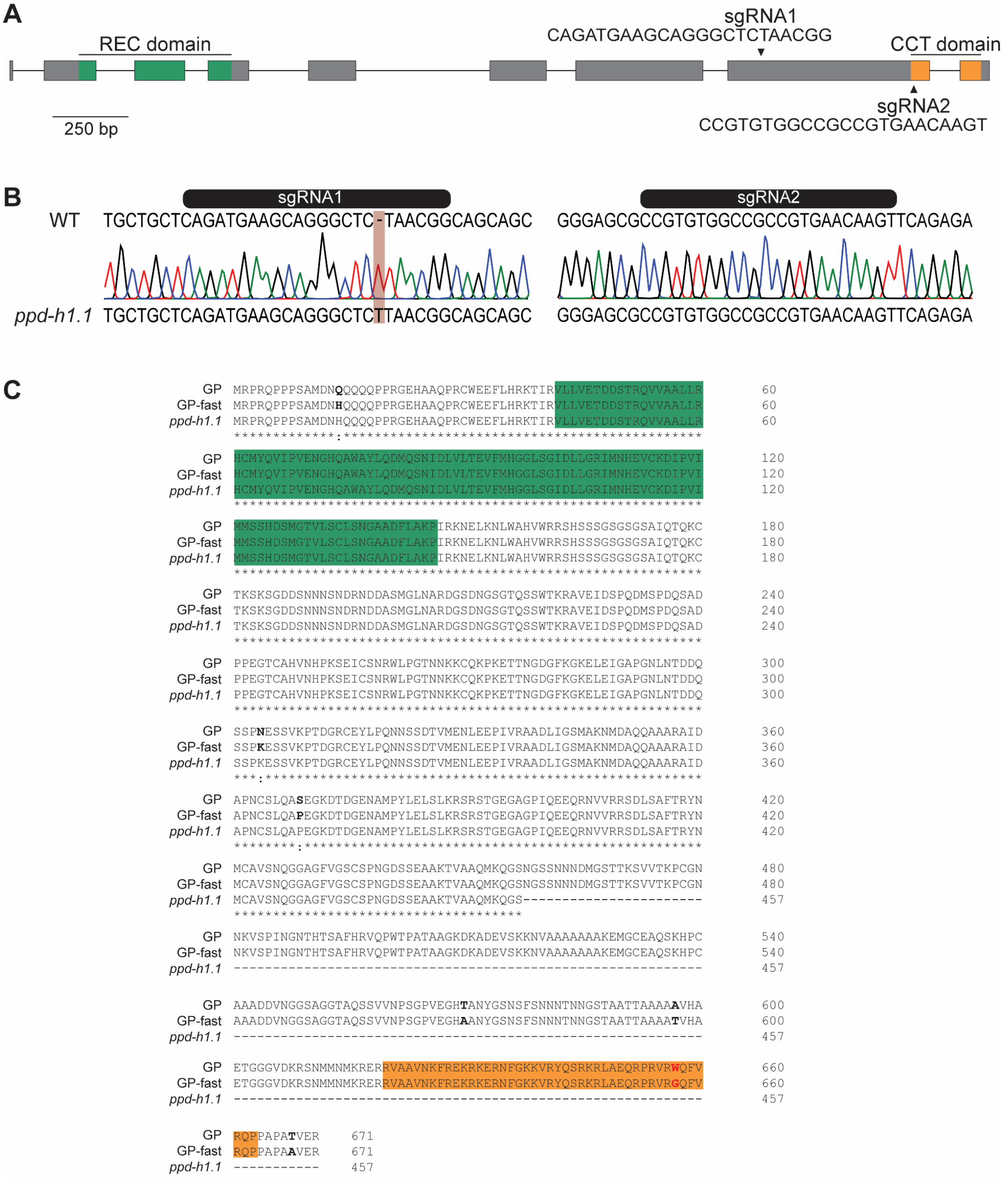

Supplementary Figure S1. Summary of CRISPR/Cas9-Induced Mutation in *Ppd-H1.* (A) Schematic overview of the *Ppd-H1* gene. Boxes indicate exons, and lines represent introns. Green and orange colors show the position of the pseudo-receiver (REC) and CONSTANS, CO-like, and TOC1 (CCT) domains, respectively, which have been identified using the NCBI conserved domain search (Marchler-Bauer et al., 2017). Two single guide RNAs (sgRNAs) used to induce mutations are indicated with arrows. (B) Sanger sequencing results of the genome-edited M1 homozygous *ppd-h1.1* mutant and the wild type (WT) GP-fast (*Ppd-H1*) at the sgRNA sites. Highlighted in red is the newly introduced mutation by sgRNA1 (T insertion at CDS position 1371 according to the gene model *HORVU.MOREX.r3.2HG0107710.1*). sgRNA2 did not introduce new mutations. (C) Multiple sequence alignment of the translated coding sequences (CDS) of the natural mutant allele *ppd-h1* from the spring barley cultivar Golden Promise (GP), of the wild-type *Ppd-H1* from its near-isogenic line GP-fast, and of the CRISPR/Cas9-induced *ppd-h1.1* in the GP-fast background using CLUSTAL Omega (1.2.4, Madeira et al., 2022). Green and orange boxes highlight the REC and CCT domains, respectively. Bold letters indicate amino acid differences between the *Ppd-H1* and *ppd-h1* allele. The amino acid change caused by SNP 22 (Turner et al., 2005) is indicated in red. Asterisks indicate positions that are shared across all sequences, colons indicate positions with conservation between groups of strongly similar properties. *ppd-h1.1* has a truncated protein of 444 amino acids due to a premature stop codon that was introduced by the 1-bp insertion.

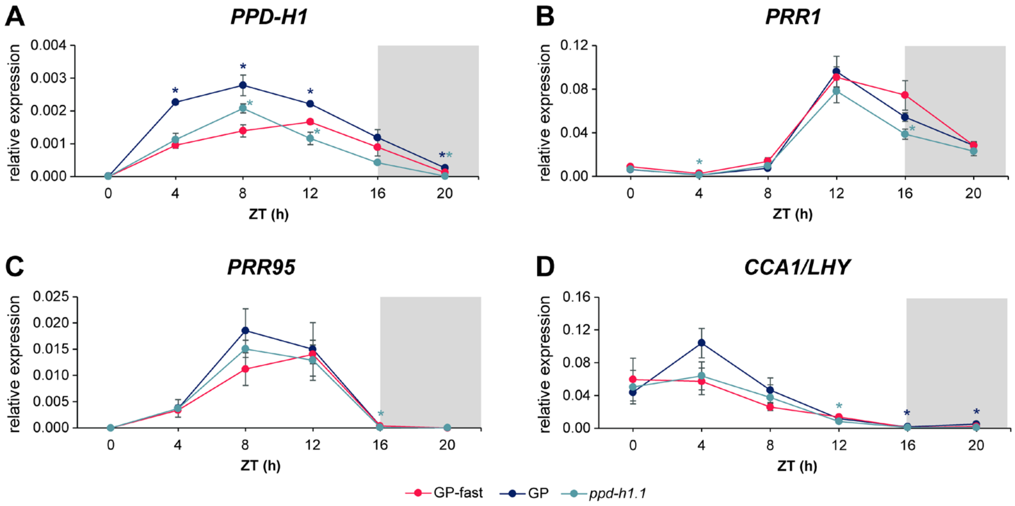

Supplementary Figure S2. Effects of *PPD-H1* on the Diurnal Gene Expression Pattern of Selected Circadian Clock Genes. (A-D) Relative expression of *PPD-H1* (A), *PRR1* (B), *PRR95* (C) and *CCA1/LHY* (D) in the spring barley cultivar Golden Promise (GP, *ppd-h1*) (dark blue), its derived near-isogenic line GP-fast (*Ppd-H1*) (red), and the CRISPR/Cas9-induced mutant *ppd-h1.1* (*ppd-h1*) (light blue). Leaf samples were taken every 4 h from Zeitgeber Time (ZT) 0 to 20. Grey overlay indicates night hours. Each value represents the mean of three independent biological replicates except for the *PPD-H1* expression in GP at ZT 4 where only two biological replicates were processed. Error bars indicate the standard deviation of the mean of three biological replicates. Student's *t*-test was conducted to compare relative expression of each gene between GP-fast and GP and between GP-fast and *ppd-h1.1*. Asterisks indicate significant differences (p < 0.05) between GP-fast and GP (light blue) and between GP-fast and *ppd-h1.1* (dark blue) at corresponding timepoints.

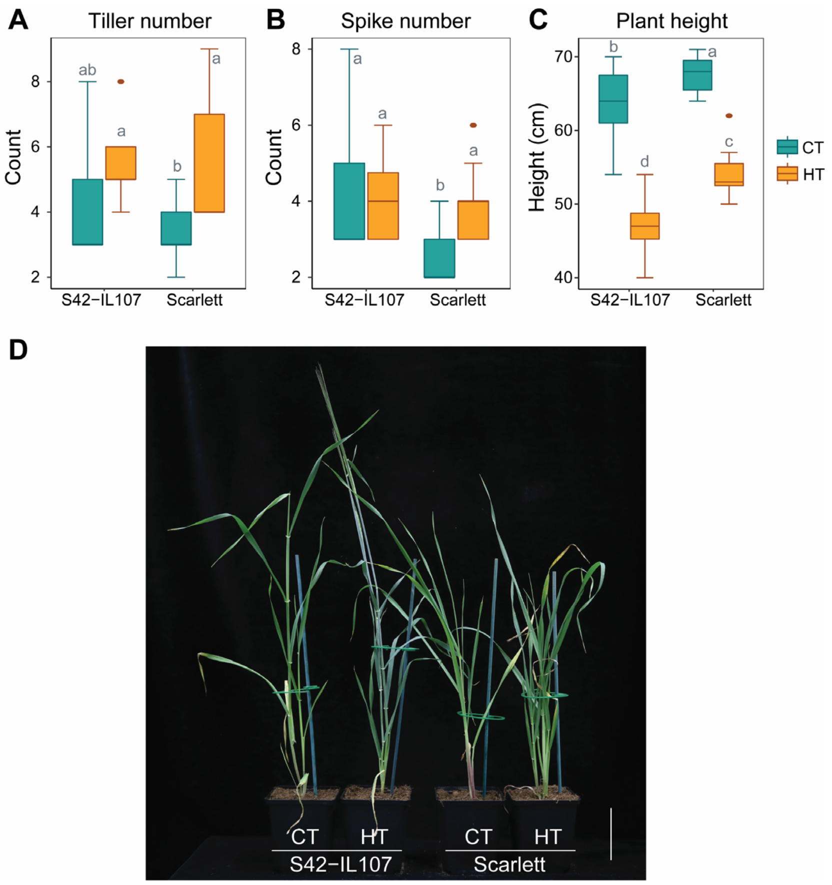

Supplementary Figure S3. Effects of High Ambient Temperature on Shoot Growth in Scarlett and S42-IL107. (A-C) Comparison of number of tillers (A) and spikes (B), and plant height (C), between control (CT, 20 °C/16 °C, day/night) and high ambient temperatures (HT, 28 °C/24 °C, day/night) in the spring barley cultivar Scarlett (*ppd-h1*) and its derived near-isogenic line (NIL) S42-IL107 (*Ppd-H1*). (D) Representative plants of Scarlett (*ppd-h1*) and its NIL S42-IL107 (*Ppd-H1*) grown under CT and HT at 32 days after emergence (DAE). Scale bar = 10 cm. Each box shows the median and interquartile range (IQR), and whisker lines extend to the smallest and largest values within 1.5*IQR from the lower and upper quartiles, respectively. Outliers beyond this range were represented by individual points. For the boxplots, statistical groups were assigned using ANOVA followed by a Tukey's post-hoc test. Different letters above the boxplots indicate significant differences between groups ($p < 0.05$). n = 10-20 plants.

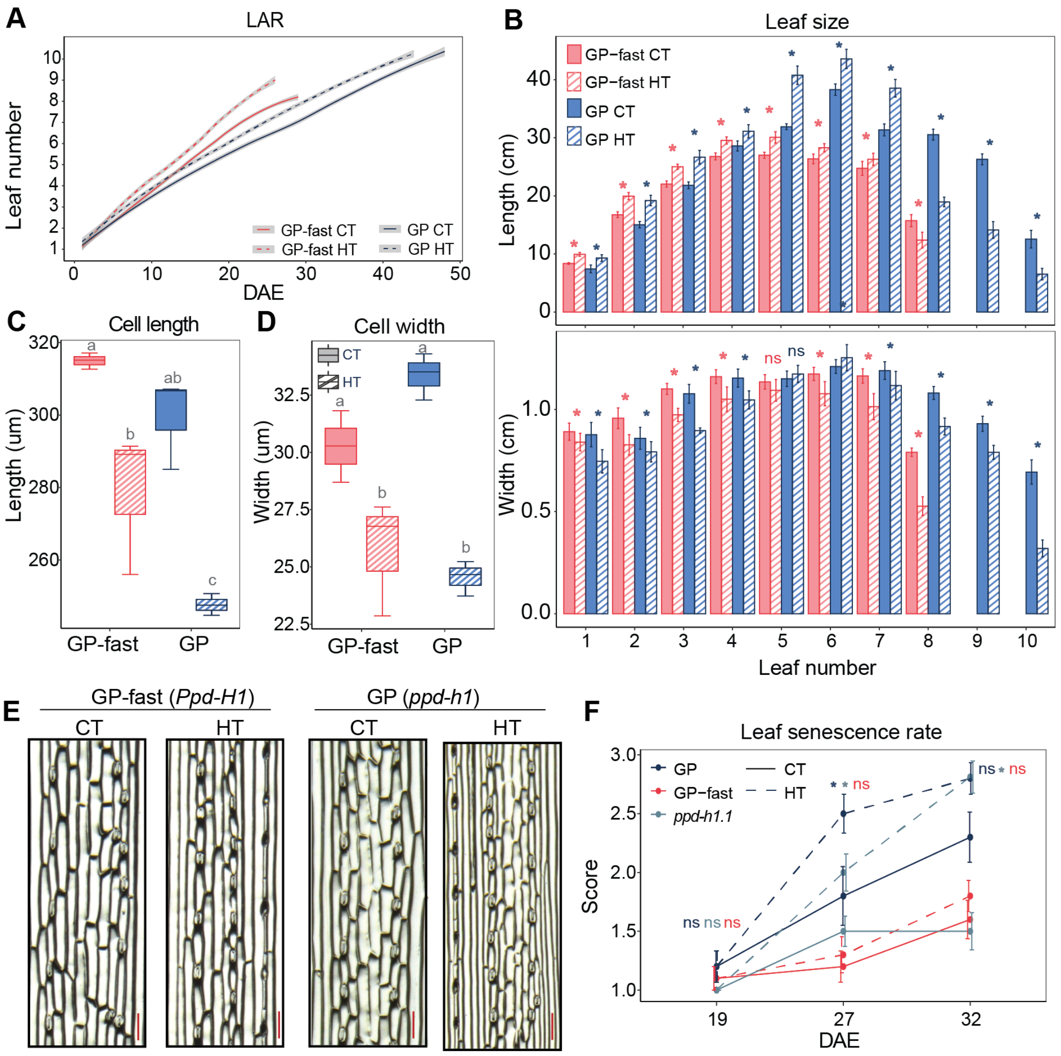

Supplementary Figure S4. Effects of High Ambient Temperature on Leaf Development and Senescence. (A) Leaf appearance rate (LAR) measured as the time interval between the sequential emergence of leaves on the main stem of the spring barley cultivar Golden Promise (GP, *ppd-h1*) and its near-isogenic line GP-fast (*Ppd-H1*) under control (CT, 20 °C/16 °C, day/night) and high ambient temperature (HT, 28 °C/24 °C, day/night). The trend line is calculated using a polynomial regression (loess smooth line), and the grey area shows the 95% confidence interval. n = 10. (B) The length (top) and width (bottom) of the leaf blade were measured when the leaf on the main culm fully expanded in GP-fast and GP under CT and HT. n = 15. Error bars indicate the standard error of biological replicates. (C-D) Comparison of cell length (C) and width (D) of the seconded fully elongated leaf (L2) among GP-fast and GP under CT and HT. Each box shows the median and interquartile range (IQR), and whisker lines extend to the smallest and largest values within 1.5*IQR from the lower and upper quartiles, respectively. Outliers beyond this range are represented by individual points. Statistical groups were assigned using ANOVA followed by a Tukey's post-hoc test. Different letters above the boxplots indicate significant differences between groups ($p < 0.05$). n ≥ 60. (E) Representative epidermal imprint images of the L2 of GP-fast and GP grown under CT and HT. Scale bar = 100 µm. (F) Leaf senescence was visually assessed based on the percentage of the non-green area on the L2 of GP-fast and GP grown under CT and HT at three time points. n = 15. Error bars indicate the standard error of 10-16 biological replicates. Student's *t*-test was conducted in B and F to compare the traits within the same genotype. Asterisks indicate significant differences ($p < 0.05$). ns represents no significance.

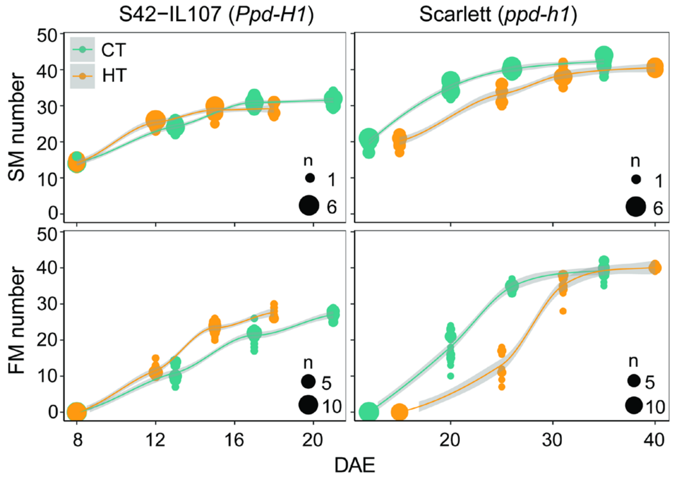

Supplementary Figure S5. Effects of High Ambient Temperature and *PPD-H1* on the Spikelet Meristem and Floral Meristem Induction Rate in Scarlett and S42-IL107. The number of spikelet meristems (SMs) (top) and floral meristems (FMs) (bottom) at W2.0, W3.5, W4.5, and W6.0 in Scarlett (*ppd-h1*) and its near-isogenic line S42-IL107 under control (CT, 20 °C/16 °C, day/night) and high ambient temperature (HT, 28 °C/24 °C, day/night). The four time points represent Waddington stage W2.0, W3.5, W4.5, and W6.0, corresponding to the days after emergence (DAE). Dot sizes indicate the number of overlapping data points. The trend line is calculated using a polynomial regression (loess smooth line), and the grey area shows the 95% confidence interval. n = 6-17.

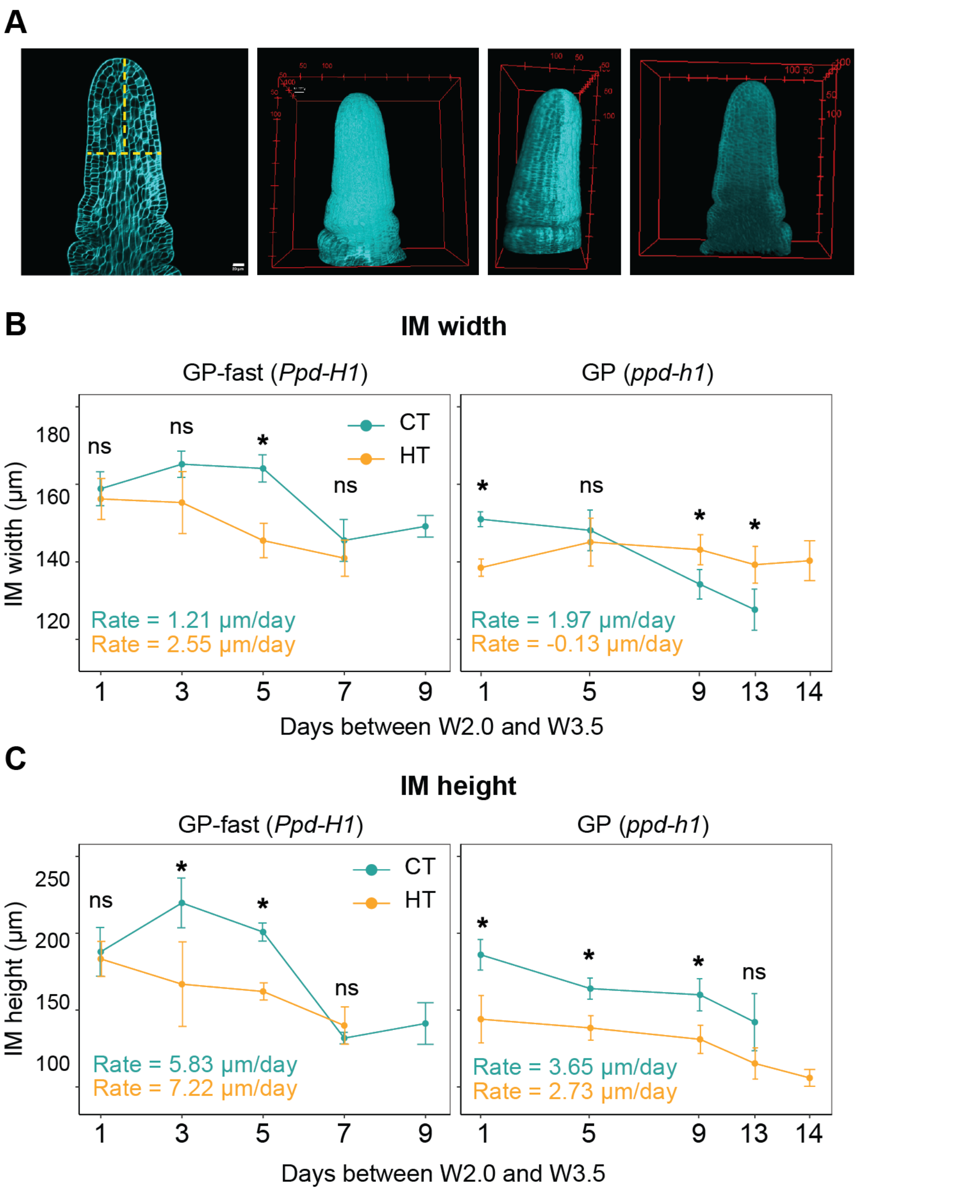

Supplementary Figure S6. Effects of High Ambient Temperature and *PPD-H1* on Inflorescence Meristem Size in GP-fast and GP. (A) Representative confocal Z-stack images of an inflorescence meristem (IM) at the W2.0 stage. The edge of IM was defined using the 3D viewer, and the width and height of IM were measured in the central section of IM. Horizontal and vertical dashed lines in the left image indicate where IM width and height were measured. Scale bar = 20 µm. (B-C) Comparison of IM width (B) and height (C) in Golden Promise (GP, *ppd-h1*) and the derived near-isogenic line GP-fast (*Ppd-H1*) under control (CT, 20 °C/16 °C, day/night) and high ambient temperature (HT, 28 °C/24 °C, day/night) from W2.0 to W3.5 by days after emergence (DAE). The x-axis indicates the corresponding days from W2.0 to W3.5 (left to right). The rate of reduction in IM width and height was calculated by dividing the changes in IM width and height by the number of days between W2.0 and W3.5. Error bars indicate the standard deviation. A Student's *t*-test was conducted to compare the IM width or height between CT and HT within the same genotype. Asterisks indicate significant differences ($p < 0.05$) between CT and the corresponding timepoint. n ≥ 3.

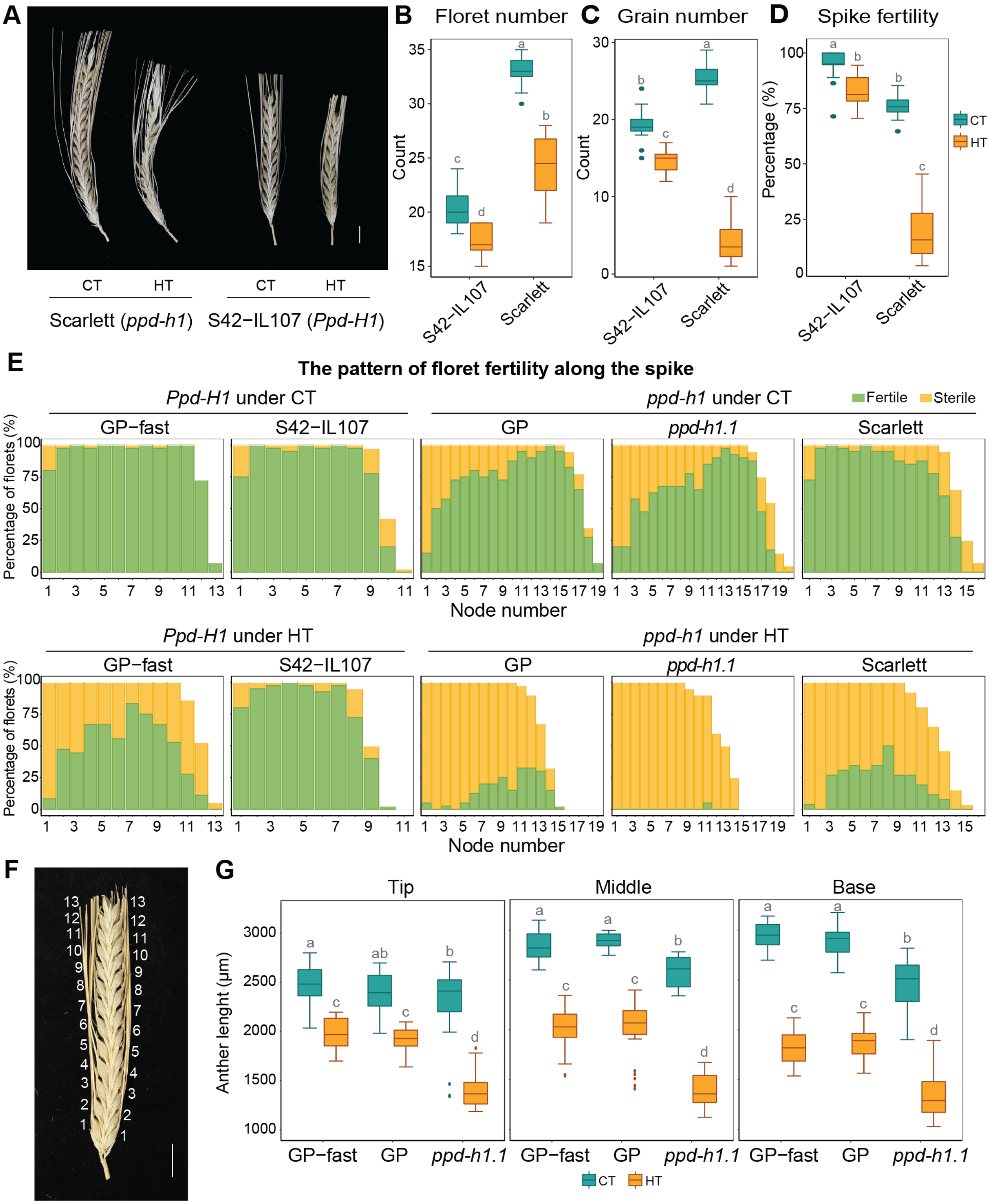

Continued on next page

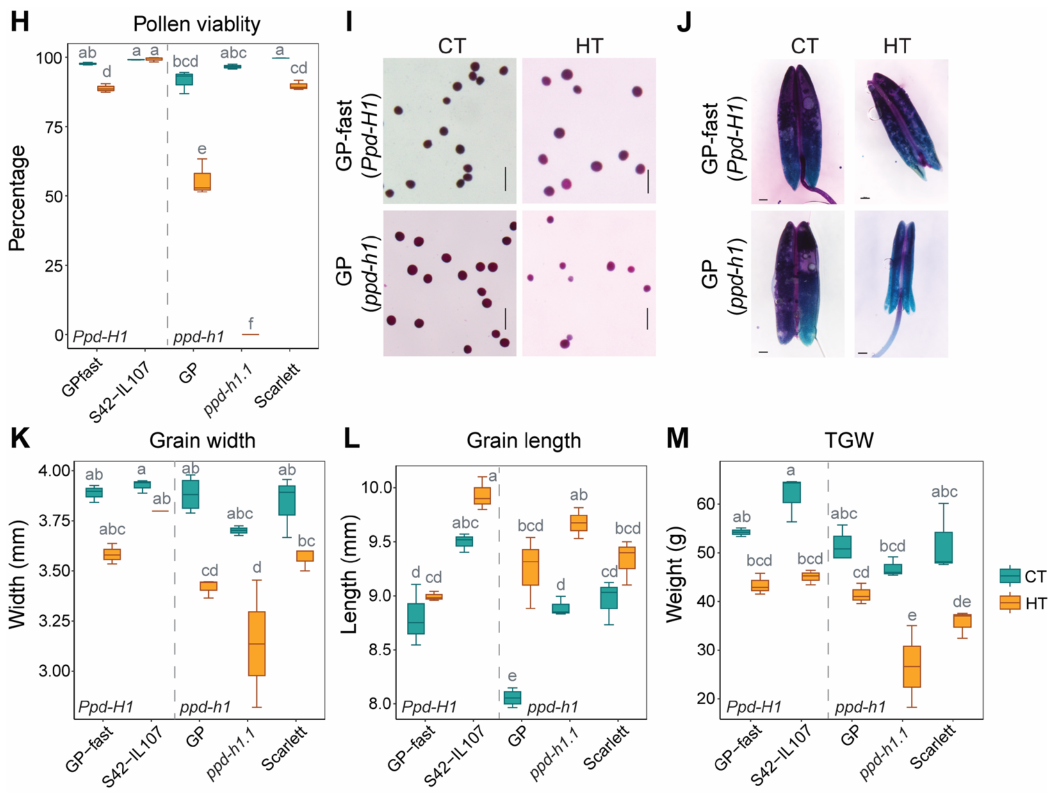

Supplementary Figure S7. Effects of High Ambient Temperature and *PPD-H1* on Spike Fertility and Grain Yield. (A) Main spike morphology of the spring barley cultivar Scarlett (*ppd-h1*) and the derived near-isogenic line S42-IL107 (*Ppd-H1*) grown under control (CT, 20°C/16 °C, day/night) and high ambient temperature (HT, 28°C/24 °C, day/night). Scale bar = 1 cm. (B-C) Number of florets (B) and grains (C) on the main shoot apex (MSA) in Scarlett and S42-IL107 under CT and HT. n = 11-15. (D) Spike fertility on the MSA was measured by the ratio of the grain number to the floret number on the MSA. n = 11-15. (E) Percentage of fertile and sterile florets at each node in mutant *ppd-h1* genotypes, Golden Promise (GP, *ppd-h1*), ppd-h1.1 (*ppd-h1*), and Scarlett (*ppd-h1*), and in wild-type *Ppd-H1* genotypes, GP-fast (*Ppd-H1*) and S42-IL107 (*Ppd-H1*). n = 20. (F) Representative image of florets at each rachis node. The floret located at the basal rachis node of the spike is numbered as 1. (G) Comparison of anther length in the florets at the tip, middle and base of the inflorescence in GP-fast (*Ppd-H1*), GP (*ppd-h1*), and p*pd-h1.1* (*ppd-h1*) under CT and HT. n ≥ 25. (H) Comparison of pollen viability in the florets at the middle of the spike among all genotypes under CT and HT. n = 3. (I-J) Representative images of pollen (I) and anthers (J) after Alexander's staining in GP-fast and GP under CT and HT. Scale bars = 100 µm (I) and 200 µm (J). (K-M) Comparison of grain length (K), width (L), and thousand-grain weight (TGW) (M) across genotype and ambient temperature. n = 3. Each box shows the median and interquartile range (IQR), and whisker lines extend to the smallest and largest values within 1.5 X the IQR from the lower and upper quartiles, respectively. Outliers beyond this range are represented by individual points. For the boxplots, statistical groups were assigned using ANOVA followed by a Tukey's post-hoc test. Different letters above the boxplots indicate significant differences between groups ($p < 0.05$).

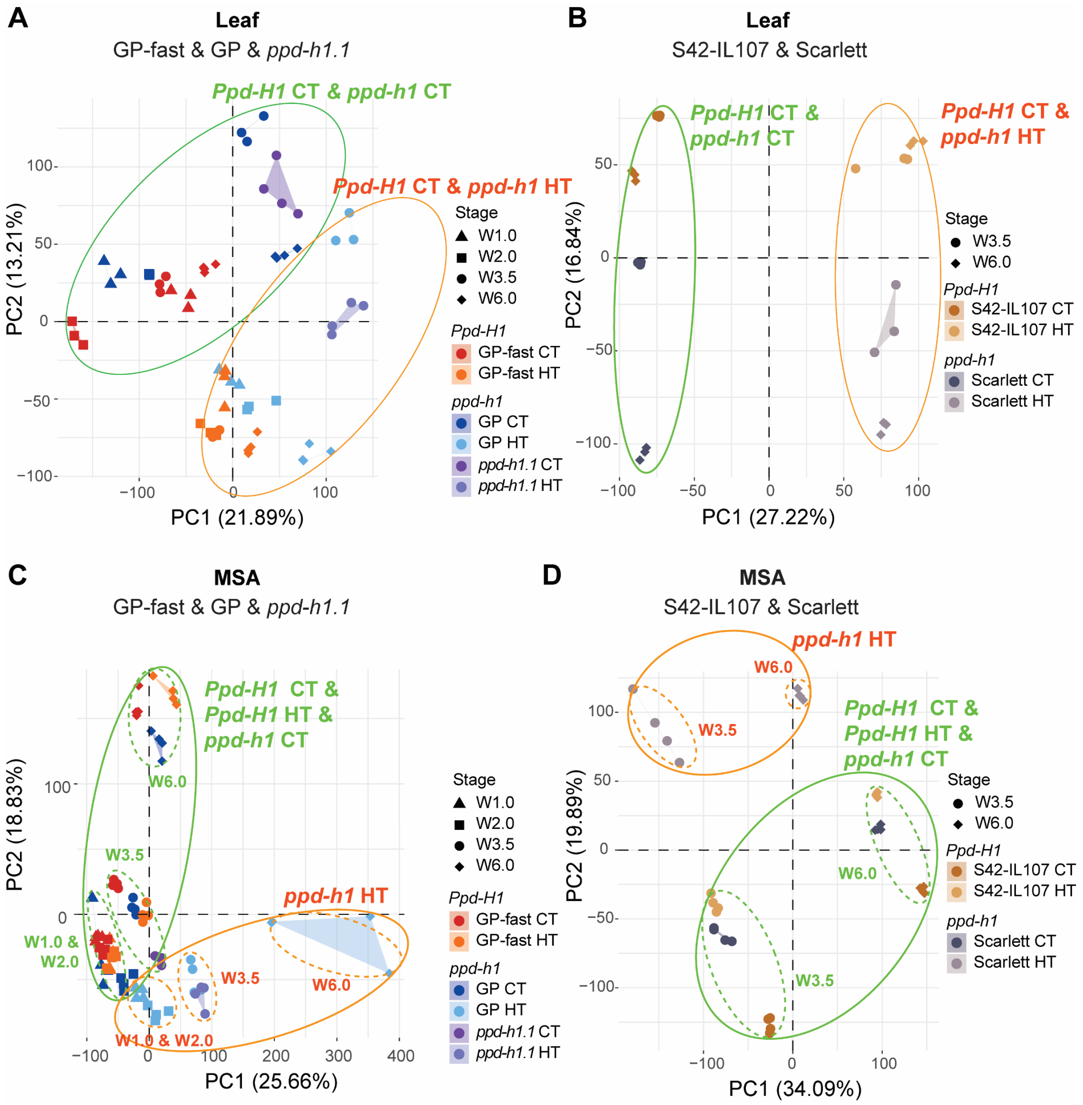

Supplementary Figure S8. Principal Component Analyses in the Leaf and Shoot Apex. (A-B) The principal component (PC) analyses in the leaf transcriptomes of spring barley cultivar Golden Promise (GP, *ppd-h1*), the derived near-isogenic line (NIL) GP-fast (*Ppd-H1*), and CRISPR-induced mutant *ppd-h1.1* (A) and of Scarlett (*ppd-h1*) and S42-IL107 (*Ppd-H1*) (B) under control (CT, 20 °C/16 °C, day/night) and high ambient temperature (HT, 28 °C/24 °C, day/night). (C-D) The PC analyses in the main shoot apex (MSA) transcriptomes of GP (*ppd-h1*), GP-fast (*Ppd-H1*), and *ppd-h1.1* (C) and of Scarlett (*ppd-h1*) and S42-IL107 (*Ppd-H1*) (D) under CT and HT. PC1 captures the highest variation, while PC2 captures the second highest. The percentage values associated with PC1 and PC2 indicate the proportion of the total variance in the dataset explained by each component.

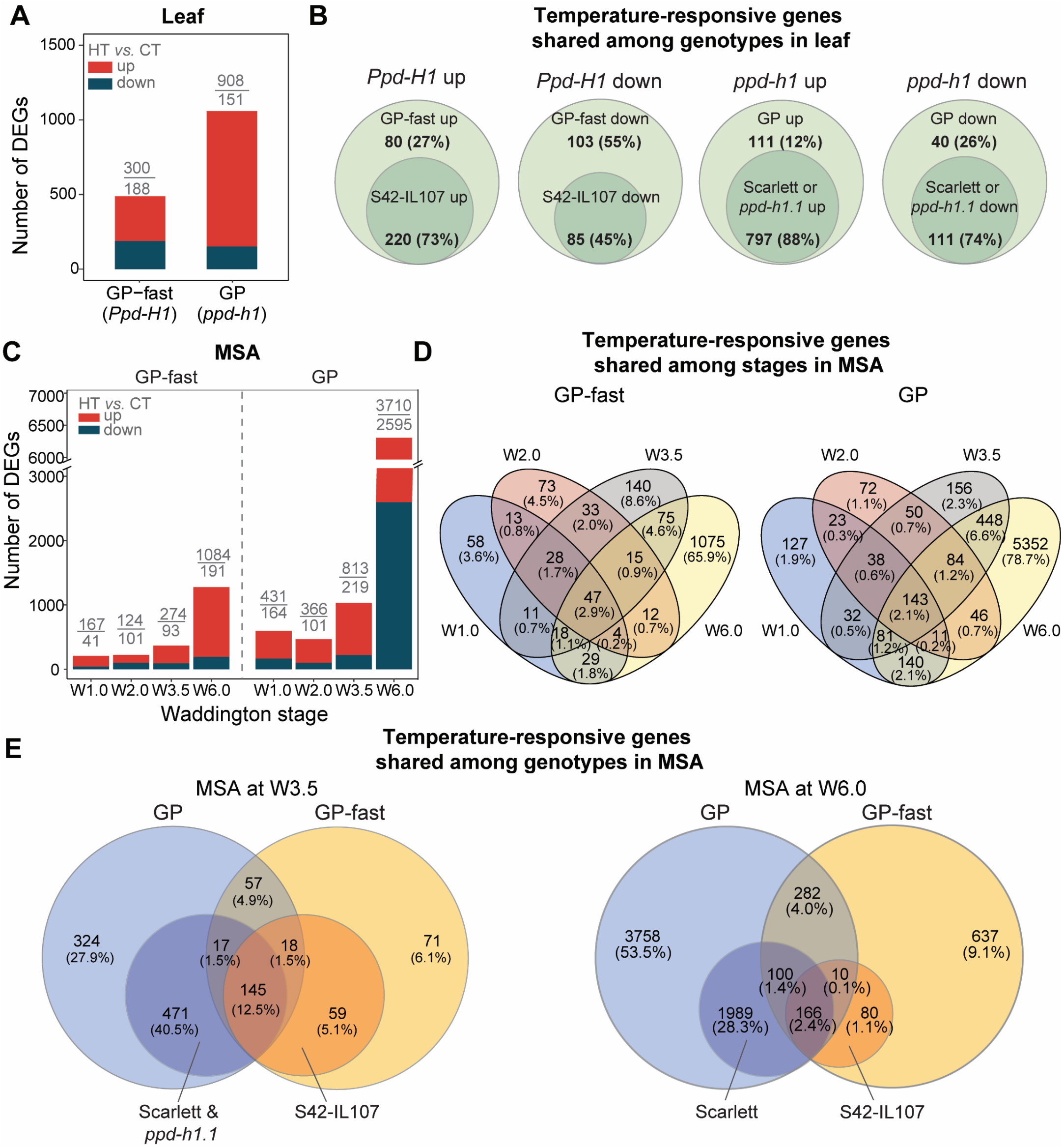

Supplementary Figure S9. Differentially Expressed Gene Identification in the Leaf and Shoot Apex. (A) Number of differentially expressed genes (DEGs) that were upregulated (red) or downregulated (dark blue) in response to high ambient temperatures (HT, 28 °C/24 °C, day/night) across at least three developmental stages in the leaf of GP-fast (Ppd-H1) (left) and Golden Promise (GP) (right). $|log_2FC| \geq 1$, BH.FDR < 0.01. n = 4. (B) Venn diagrams represent the overall temperature-responsive DEGs shared between GP-fast and S42-IL107, and between GP and Scarlett or *ppd-h1.1* in the leaf samples. (C) Number of DEGs that were upregulated (red) or downregulated (dark blue) in response to HT at each developmental stage in the main shoot apex (MSA) of GP-fast (left) and GP (right). $|log_2FC| \geq 1$, BH.FDR < 0.01. n = 4. (D) Venn diagrams represent the temperature-responsive DEGs in the MSA shared among four developmental stages in GP-fast (left) and GP (right), respectively. (E) Venn diagrams indicate the temperature-responsive DEGs in the MSA shared among all genotypes at W3.5 (left) and W6 (right), respectively. For the gene filtering and statistics, see Supplementary Dataset 2 and Supplementary Dataset 3.

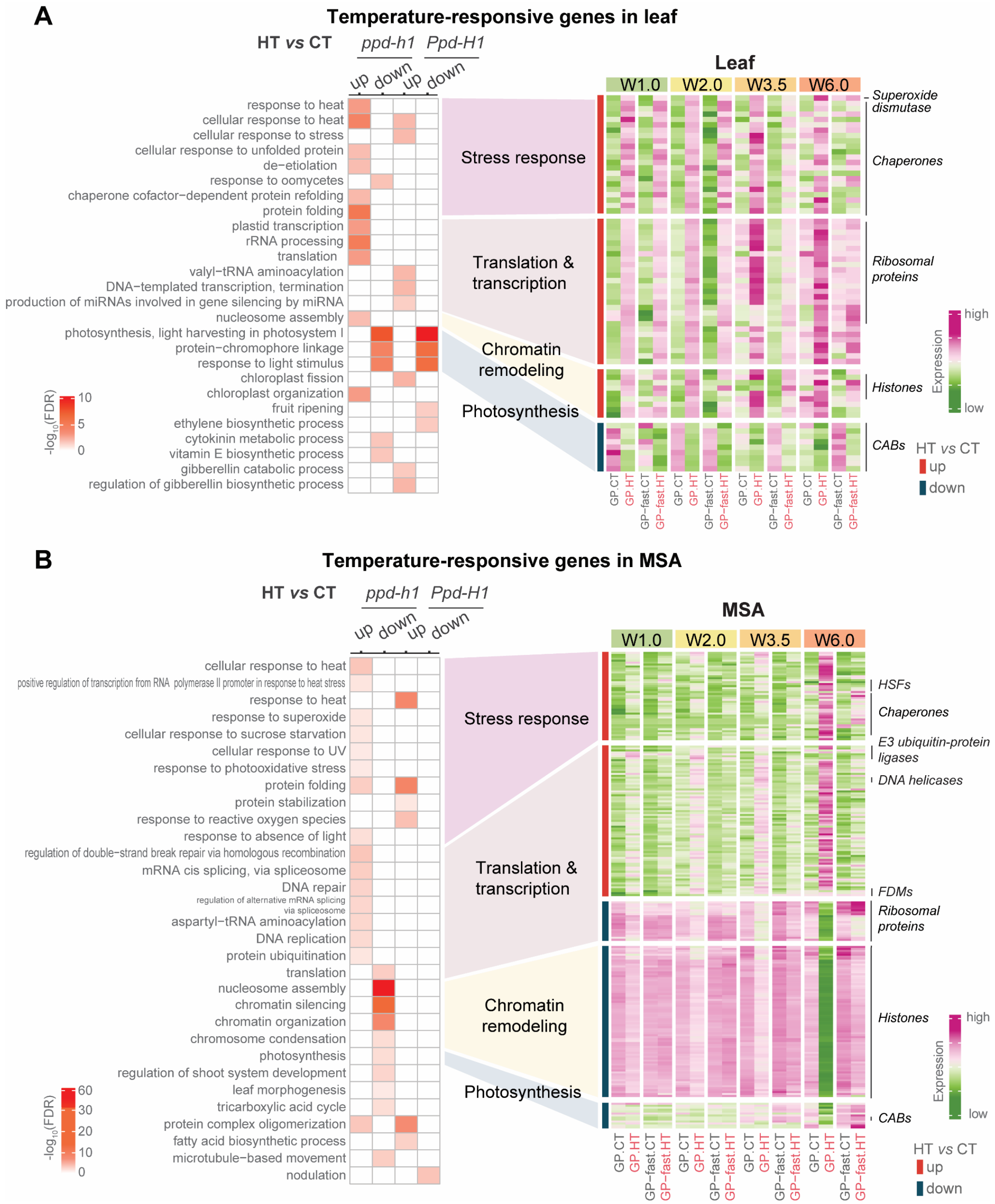

Supplementary Figure S10. Function and Expression Pattern of Temperature-Responsive Genes in the Leaf and Shoot Apex. (A-B) Gene Ontology (GO) analysis of the upregulated and downregulated differentially expressed genes (DEGs) in response to high ambient temperature (HT, 28 °C/24 °C, day/night) in the leaf (A) and main shoot apex (B) of the wild-type *Ppd-H1* and mutant *ppd-h1* genotypes. Heatmaps of GO enrichment (left) represent the significance of the top 10 GO terms, and the heatmaps of the gene expression (right) represent the DEGs that belong to the interested GO terms. The colour scale represents the range of Z-score normalized mean transcript per million (TPM) values of three to four biological replicates. The darker green hues represent lower expression, while darker magenta hues represent higher expression relative to other samples in the same gene. The representative gene families are labelled on the side of the heatmap. For the GO terms, gene list, and the TPM values, see Supplemental Dataset 4 and Supplemental Dataset 5.

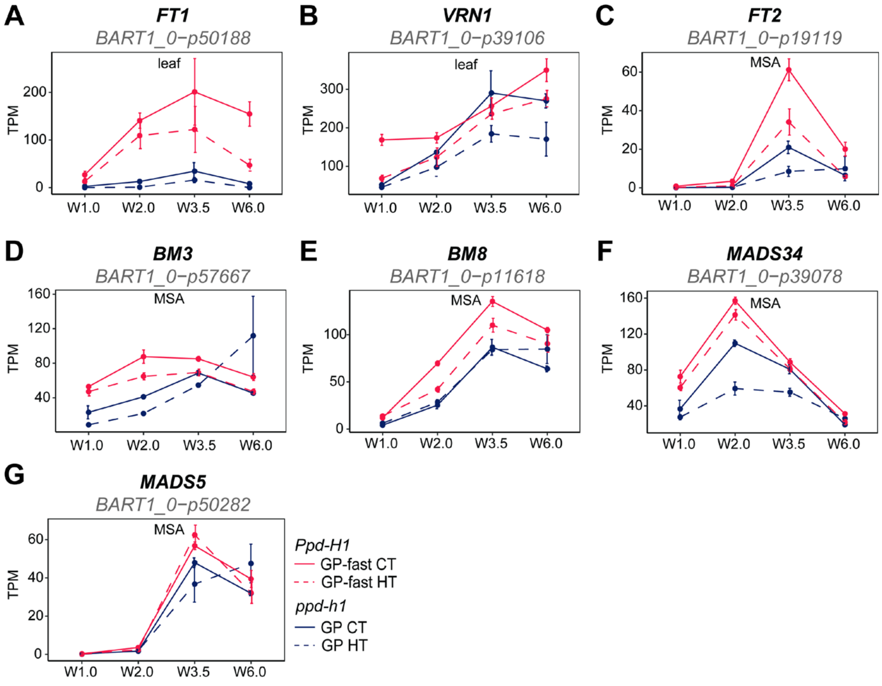

Supplementary Figure S11. Expression of Known Flowering Time and Flower Development Regulators in the Leaf and Shoot Apex. Gene expression patterns of *FT1* (A) and *VRN1* (B) in the leaf and *FT2* (C), *BM3* (D), *BM8* (E), *MADS34* (F), and *MADS5* (G) in the main shoot apex (MSA) from RNA-sequencing in spring barley cultivar Golden Promise (GP, *ppd-h1*) and its near-isogenic line GP-fast (*Ppd-H1*) under control (CT, 20 °C/16 °C, day/night) and high ambient temperatures (HT, 28 °C/24 °C, day/night). Transcript levels are shown in transcripts per million (TPM). The line plots represent the gene expression related to known flowering time and floral development regulators in GP-fast (red) and GP (dark blue) under CT (solid line) and HT (dashed line). Error bars indicate the standard deviation of three to four biological replicates. *FT1*, *FLOWERING LOCUS T1*; *VRN1*, *VERNALIZATION 1*. *FT2*, *FLOWERING LOCUS T2*; *BM3*, *BARLEY MADS-BOX 3*; *BM3*, *BARLEY MADS-BOX 8*.

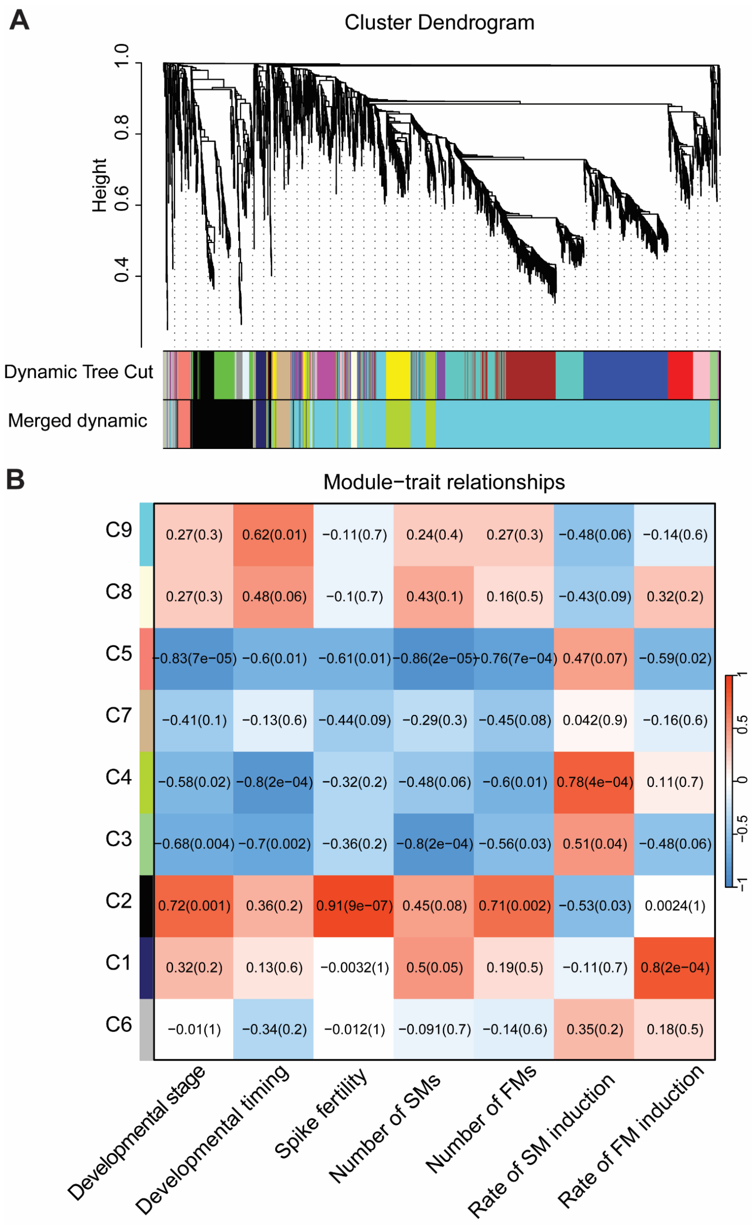

Supplementary Figure S12. Weighted Gene Co-Expression Network Analysis (WGCNA) of the Temperature-Responsive Genes in the Shoot Apex. (A) Gene cluster dendrograms of WGCNA analysis. The major tree branches into modules with different colours. The initial modules were identified by the Dynamic Tree Cut algorithm. Merged dynamic refers to the further step if the initial modules are highly similar. (B) Module-Trait correlation. Each cell represents the correlation coefficient between a module eigengene (a representative gene expression profile of a module) and a specific trait, with a corresponding *p*-value provided in parenthesis. The colour scale on the right indicates the strength and direction of the correlation. Positive correlations are shown in red hues, while negative correlations are in blue hues. The intensity of the colour reflects the magnitude of the correlation coefficient, with darker shades representing stronger correlations.

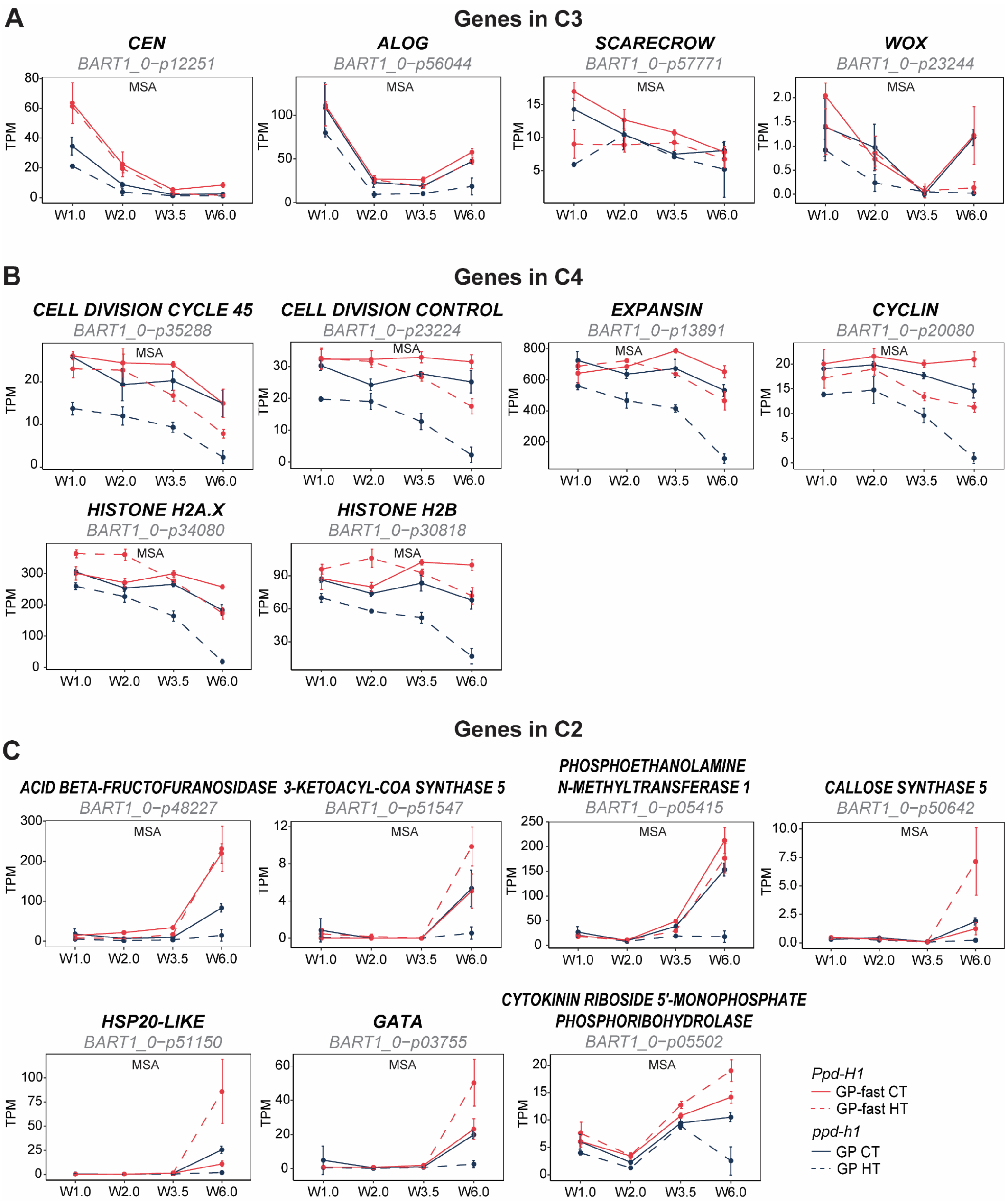

Supplementary Figure S13. The expression of Representative Genes from the Development-Related Clusters in the Shoot Apex. Transcript levels of representative genes in the development-related clusters in the main shoot apex (MSA) from the RNA-sequencing in spring barley cultivar Golden Promise (GP, *ppd-h1*) and its near-isogenic line GP-fast (*Ppd-H1*) under control (CT, 20 °C/16 °C, day/night) and high ambient temperatures (HT, 28 °C/24 °C, day/night) at vegetative (W1.0), spikelet induction (W2.0), stamen primordium (W6.0) and style primordium stages (W6.0). Transcript levels are shown in transcripts per million (TPM). The line plots represent the gene expression related to clusters C3 (A), C4 (B), and C2 (C) in GP-fast (red) and GP (dark blue) under CT (solid line) and HT (dashed line). Error bars indicate the standard deviation of four biological replicates. *CEN, CENTRORADIALIS*; *ALOG*, *ARABIDOPSIS LIGHT-DEPENDENT SHORT HYPOCOTYLS 1 (LSH1) AND ORYZA G1*; *WOX, WUSCHEL-LIKE HOMEOBOX; HSP20-LIKE, HEAT SHOCK PROTEIN 20-LIKE*.

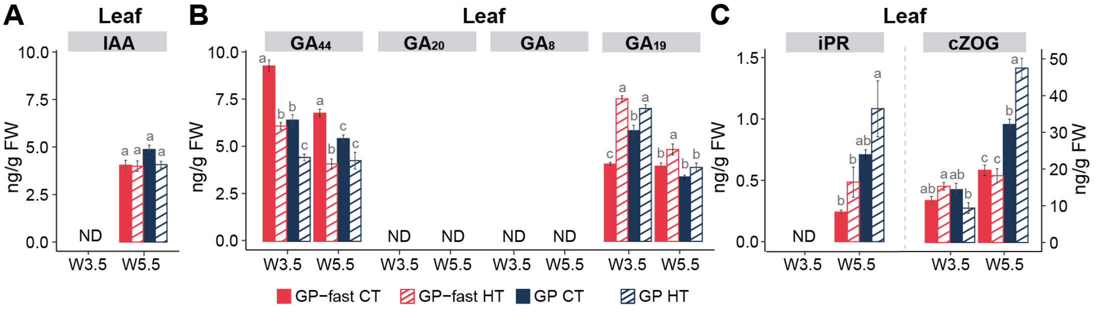

Supplementary Figure S14. Effects of *PPD-H1* and High Ambient Temperature on Auxin, Gibberellin, and Cytokinin Levels in the Leaf. (A-C) Comparison of the levels of the developmental-related phytohormones, indole-3-acetic acid (IAA) (A), gibberellin (GA) (B), and cytokinin (C) in the leaf of the spring barley cultivar Golden Promise (GP, *ppd-h1*) and its near-isogenic line GP-fast (*Ppd-H1*) grown under control (CT, 20 °C/16 °C, day/night) and high (HT, 28 °C/24 °C, day/night) ambient temperatures at W3.5 and W5.5, respectively. Error bars indicate the standard error of four biological replicates. A vertical dashed line separates the hormone groups to indicate which y-axis applies to them. The y-axis on the right corresponds to cZOG. Statistical groups were assigned using ANOVA followed by a Tukey's post-hoc test for each phytohormone per developmental stage, genotype, and condition. Different letters above the bars indicate significant differences between groups ($p < 0.05$). iPR, N6-isopentenyladenosine; cZOG, *cis*-zeatin-O-glucoside.

Supplementary Table S1. Effects of High Ambient Temperature on Grain Set and the Rate of Spikelet Meristem and Floral Meristem Induction.

Traits	Stage	GP-fast (*Ppd-H1*)			GP (*ppd-h1*)			*ppd-h1.1* (*ppd-h1*)			S42-IL107 (*Ppd-H1*)			Scarlett (*ppd-h1*)		
		**CT**	**HT**	***p***	**CT**	**HT**	***p***	**CT**	**HT**	***p***	**CT**	**HT**	***p***	**CT**	**HT**	***p***
Number of SMs	W2.0	14 ±1	15 ±1	**	18 ±1	19 ±1	*	15 ±0	14 ±1	ns	14 ±1	14 ±1	ns	20 ±1	20 ±2	ns
	W3.5	26 ±1	27 ±1	ns	39 ±2	31 ±2	****	35 ±1	34 ±4	ns	24 ±1	25 ±1	*	35 ±2	33 ±2	*
	W4.5	31 ±1	31 ±1	ns	43 ±2	35 ±1	****	47 ±1	40 ±2	ns	31 ±1	29 ±2	***	40 ±1	39 ±2	*
	W6.0	34 ±1	30 ±1	****	43 ±2	36 ±2	****	47 ±1	43 ±0	*	32 ±1	29 ±2	*	42 ±2	41 ±1	**
Number of FMs	W3.5	9 ±2	10 ±2	ns	17 ±3	11 ±3	****	15 ±1	16 ±7	ns	11 ±2	12 ±1	ns	18 ±4	13 ±4	**
	W4.5	20 ±1	25 ±4	****	31 ±2	27 ±6	*	37 ±2	36 ±1	ns	21 ±2	23 ±2	**	35 ±1	35 ±3	ns
	W6.0	31 ±1	29 ±1	***	40 ±2	35 ±2	****	43 ±1	40 ±0	*	27 ±1	28 ±2	ns	39 ±2	40 ±1	ns
Duration (days)	W2.0-W3.5	8	6		12	13		12	13		5	4		8	10	
	W3.5-W4.5	5	4		4	8		7	6		4	3		6	6	
	W4.5-W6.0	5	4		10	14		6	7		4	3		9	9	
SM induction rate (SMs/day)	W2.0-W4.5	1.3	1.6		1.6	0.8		1.7	13		1.9	2.1		1.4	1.1	
FM induction rate (SMs/day)	W3.5-W6.0	1.7	2.1		1.6	1.0		1.7	1.3		2.1	2.8		1.7	1.6	

Floret number	Maturity	21 ±2	19 ±3	**	30 ±2	22 ±3	****	35 ±2	1.5 ±4	****	20 ±2	17 ±1	****	33 ±1	24 ±3	****
Grain number	Maturity	20 ±2	15 ±2	****	24 ±3	5 ±3	****	23 ±3	25 ±0		19 ±2	15 ±2	****	25 ±2	5 ±3	****
Spike fertility (%)	Maturity	1.0 ±0	0.8 ±0.1	****	0.8 ±0.1	0.2 ±0.1	****	0.7 ±0.1	0 ±0	****	0.9 ±0.1	0.8 ±0.1	***	0.8 ±0.1	0.2 ±0.1	****
Florets/SM		0.6	0.6		0.7	0.6		0.7	0.6	****	0.6	0.6		0.8 ±0.1	0.2 ±0.1	****
Grains/SM		0.6	0.5		0.5	0.1		0.5	0		0.6	0.5		0.6	0.1	
Florets/FM		0.7	0.6		0.7	0.6		0.8	0.6		0.8	0.6		0.8	0.6	
Grains/FM		0.6	0.5		0.6	0.2		0.5	0		0.7	0.5		0.6	0.1	

Statistic test: Student *t*-test, the pairwise comparisons are conducted between control and high ambient temperature in GP-fast, GP, *ppd-h1.1*, Scarlett, and S42-IL107, respectively.

p value: $p > 0.05$ (ns); $p \leq 0.05$ (*); $p \leq 0.01$ (**); $p \leq 0.001$ (***); $p \leq 0.0001$ (****)

CT: control ambient temperature (20 °C/16 °C, day/night)

HT: high ambient temperature (28 °C/24 °C, day/night)

SM: spikelet meristem

FM: floral meristem

Supplementary Table S2. RT-qPCR Primers Used in This Study.

Target gene	Primer	Sequence 5'-3'	Size (bp)
ACTIN (*HORVU.MOREX.r3.5HG0457850*)	Forward	CGTGTTGGATTCTGGTGATG	208
	Reverse	AGCCACATATGCGAGCTTCT	
PPD-H1 (*HORVU.MOREX.r3.2HG0107710*)	Forward	GATGGATTCAAAGGCAAGGA	172
	Reverse	GAACAATTGGCTCCTCCAAA	
PRR1 (*HORVU.MOREX.r3.6HG0595250*)	Forward	GAGCATAGCATGGCACTTCA	237
	Reverse	TGTCTTTCCTCGGAAATTGG	
PRR59 (*HORVU.MOREX.r3.6HG0595250*)	Forward	GAAATTCCGCATGAAAAGGA	148
	Reverse	TTCCGCATCTTCTGTTGTTG	
CCA1/LHY (*HORVU.MOREX.r3.7HG0699010*)	Forward	CCTGGAATTGGAGATGGAGA	210
	Reverse	TGAGCATGGCTTCTGATTTG	

The fragment size is based on the genomic DNA.

Supplementary Table S3. Unlabelled Standards and Isotopically Labelled Internal Standards (I.S.) for Phytohormone Analysis.

Phytohormone	$[M-H]^-$ / $[M+H]^+$	Standard cat. no.	Phytohormone I.S.	I.S. $[M-H]^-$ / $[M+H]^+$	I.S. cat. no.
GA_8	$[M-H]^-$ = 363.145	012 265	d_2-GA_{19}	$[M-H]^-$ = 363.178	032 269
GA_{19}	$[M-H]^-$ = 361.166	012 269	d_2-GA_{19}	$[M-H]^-$ = 363.178	032 269
GA_{20}	$[M-H]^-$ = 331.155	012 248	d_2-GA_{20}	$[M-H]^-$ = 333.168	032 248
GA_{44}	$[M-H]^-$ = 345.171	012 236	d_2-GA_{44}	$[M-H]^-$ = 347.183	032 236
(±)*cis*,*trans*-ABA	$[M-H]^-$ = 263.129	013 270	d_6(+)*cis,trans*-ABA	$[M-H]^-$ = 269.167	034 272
SA	$[M-H]^-$ = 137.024	84210	d_4-SA	$[M-H]^-$ = 141.050	037 658
I3CA	$[M-H]^-$ = 162.055	003 172	d_5-IAA	$[M+H]^+$ = 181.102	031 153
IAA	$[M+H]^+$ = 176.071	45533	d_5-IAA	$[M+H]^+$ = 181.102	031 153
cZOG	$[M+H]^+$ = 382.172	001 339	d_5-tZOG	$[M+H]^+$ = 387.203	030 512
IPR	$[M+H]^+$ = 336.167	001 017	d_6-iPR	$[M+H]^+$ = 342.204	030 017

Abbreviations: (±)*cis*,*trans*-abscisic acid ((±)*cis*,*trans*-ABA), salicylic acid (SA), indole-3-acetic-acid (IAA), indole-3-carboxylic acid (I3CA), gibberellin A8 (GA_8), gibberellin A19 (GA_{19}), gibberellin A20 (GA_{20}), gibberellin A44 (GA_{44}), *cis*-zeatin-O-glucoside (cZOG),d_5-*trans*-zeatin-O-glucoside ([2H5]-tZOG), N6-isopentenyladenosine (iPR).
